# Self-resilience as a protective factor against development of post-traumatic stress disorder symptoms in police officers

**DOI:** 10.1186/s40557-016-0145-9

**Published:** 2016-10-17

**Authors:** Jong-Ku Lee, Hyeon-Gyeong Choi, Jae-Yeop Kim, Juhyun Nam, Hee-Tae Kang, Sang-Baek Koh, Sung-Soo Oh

**Affiliations:** 1 Department of Occupational and Environmental Medicine, Wonju Severance Christian Hospital, Wonju College of Medicine, Yonsei University, 20 ilsan-ro, Wonju, Gangwon 220-701 South Korea; 2 Department of Preventive Medicine, Wonju College of Medicine, Yonsei University, 20 ilsan-ro, Wonju, 220-701 Gangwon South Korea

**Keywords:** Police officer, Post-traumatic stress disorder symptoms, Job stress, Resilience

## Abstract

**Background:**

This study was conducted to check whether self-resilience, one of the characteristics known to affect the occurrence of post-traumatic stress disorder (PTSD) symptoms after experiencing traumatic events, could serve as a protective factor for police officers whose occupational factors are corrected.

**Methods:**

We conducted a cross-sectional study in which 112 male police officers in Gangwon Province participated. They visited the Wonju Severance Christian Hospital Occupational Environment Center for medical check-ups from June to December 2015. Their general characteristics were identified using structured questionnaires, and they were asked to fill in the Korean Occupational Stress Scale-Short Form (KOSS-SF). Further, the Center for Epidemiologic Studies-Depression Scale (CES-D), Connor–Davidson Resilience Scale-Korean (CD-RI-K), and Impact of Event Scale-Revised-Korean version (IES-R-K) were used to evaluate their job stress, depression, self-resilience, and PTSD symptoms. Logistic regression analysis was conducted to correct their personal, occupational, and psychological factors to analyze the relationship between self-resilience and PTSD symptoms.

**Results:**

Among 112 respondents who experienced a traumatic event, those with low self-resilience had significantly higher rate of PTSD symptoms than those with high self-resilience even after correcting for the covariate of general, occupational, and psychological characteristics (odds ratio [OR] 3.51; 95 % CI: 1.06–19.23).

**Conclusions:**

Despite several limitations, these results suggest that a high degree of self-resilience may protect police officers from critical incident-related PTSD symptoms.

## Background

Police officers are exposed to various traumatic and stressful events such as violence, witnessing traffic accidents, and life-threatening events [1]. The number of people per police officer in Korea as of 2015 was 426, which was quite higher than that in advanced countries, and the occurrence of violent crimes has increased. Some police officers who experience a traumatic event might develop post-traumatic stress disorder (PTSD) symptoms [2]. Further, it has been reported that prevalence rates of PTSD symptoms among police officers are between 7 and 19 % [1, 3].

However, not everyone who experiences a traumatic event develops PTSD symptoms. In particular, the risk level of PTSD symptoms among police officers is diverse [4]. Individual adaptability to traumatic events or stressful situations can serve as a psychological factor related to the prevention of PTSD symptoms. The term self-resiliency was first used by Rutter in 1985 [5] after he discovered that some people easily adapt to environmental difficulties and stressful situations, and has been used since. While resilient people can easily adapt to stressful situations, non-resilient people become impulsive and threatening; they overly control their demands and impulses, feel anxiety, and show signs of non-adaptability. In a study conducted on college students to examine the relationship between self-resilience and adaptability, it was observed that those with resilience, good interpersonal skills, and an ability to control their emotions had lower levels of anxiety and aggression compared to those who did not have these abilities. Self-resilience has positive effects on mental health [6]. Resilient people feel less stressed, are less lonely, have better social adaptation skills, and experience greater psychological comfort [7]. Based on these findings, it is determined that self-resilience-related factors lower maladjustment and affect the occurrence of PTSD symptoms in those who have experienced a traumatic event directly or indirectly.

Numerous studies have been conducted on protective factors for PTSD symptoms in people with special occupation. Besides the most salient predictor of PTSD symptoms, which is the nature of the traumatic event per se, three other risk factors were consistently identified across studies in a meta-analysis by Brewin et al. [8]: psychiatric history, family history, and mental disorders. In addition, personality traits (for example, hostility, neuroticism, positive world assumptions, and better social functioning) were also identified as predictors of PTSD symptoms [8–10]. However, relatively few studies have been conducted on the relationship between self-resilience and the occurrence of PTSD symptoms. Most of studies have been focused on firefighters. In particular, there are almost no studies related to resilience mediated mental health of police officers conducted by correcting for their occupational factors. However, there are differences between firefighters and police officers with respect to the intensity, frequency, and types of cases that they handle. It is generally known that firefighters face more intense cases, and as such, most of the studies to date have focused mostly on firefighters. Even if their degree of exposure is considered to be relatively mild, police officers who experience various forms of traumatic events can certainly be considered a high risk group for PTSD, and since studies on police officers are almost non-existent, additional studies on them are deemed necessary.

Therefore, this study was conducted on police officers in the Gangwon area, who experienced traumatic events, to examine whether self-resilience could serve as a protective factor against the occurrence of PTSD symptoms. Their general, occupational, and psychological characteristics were corrected for, and the relationship between self-resilience and the occurrence of PTSD symptoms was examined.

## Methods

### Study subjects

This cross-sectional study involved police officers from 15 regional police departments (Wonju, Sokcho, Hwacheon, Hoengsung, Yanggu, Chuncheon, Pyeongchang, Jungsun, Gangneung, Yeongwol, Donghae, Inje, Goseong, Hongcheon, and Taebaek) in Gangwon from June 4 to December 20, 2015. The study was initially conducted on 272 police officers among 3461 police officers in Gangwon, by convenience sampling, study subjects who have not experienced traumatic events for the past 6 months and carried out rotation duties between patrol job and office work. They were all aged over 49 years, have been performing rotation duties between patrol and desk job for more than 20 years, but all of them were engaged in patrol jobs as the present task. All of them visited the Department of Occupation and Environmental Medicine, Wonju Christian Hospital, for routine health check-ups, special screening for work-shifts, PTSD symptoms, and depression. All participants completed a self-reported questionnaire for evaluating job-related stress, depression, self-resilience, and PTSD symptoms. Among 272 participants who completed the survey, answers of 39 were incorrect, and 13 were previously diagnosed with mental disorders or had familiar history of mental disorders; we excluded such participants. Among the 220 remaining police officers, 112 had experienced traumatic events and were finally enrolled as study subjects. Written informed consent was obtained from participants prior to their involvement in the study.

### General characteristics

Basic demographical information was collected by self-reported questionnaires that included the following: age range, 47 to 60 years (the data were processed as continuous data), education status (middle school or below, high school, or college or above), marital status (married, divorced, or widowed), smoking status (non-smoker, ex-smoker, or current smoker), and frequency of drinking alcohol (none, ≤ one drink per week, or ≥ two drinks per week).

### Occupational characteristics

The occupational characteristics surveyed included the following: service area (urban or rural); years of patrol service (<10 year, 10–19 years, ≥ 20 years), and job stress (measured using the Korean Occupational Stress Scale-Short Form [KOSS-SF]).

#### KOSS-SF

Twenty-four questions on the 4-point Likert scale from 1 (“not at all”) to 4 (“very much”) were included in the KOSS-SF, which was validated using factor analysis and standardized validation process by the National Study for Development and Standardization of Occupational Stress and included the following seven subscales: job demand (four items), insufficient job control (four items), inadequate social support (three items), job insecurity (two items), organizational system (four items), lack of reward (three items), and occupational climate (four items). The sum of the conversion scores for each of the seven sectors was divided by seven to obtain the total occupational stress score. Based on the reference values of occupational stress for men (Short Form) [11], the total study groups were categorized by quartile to observe the score distribution of occupational stress, and the data were processed as continuous data to conduct *t*-test and logistic analysis.

### Psychosocial characteristics

To evaluate the subjects’ psychosocial levels, the Korean Center for Epidemiologic Studies Depression Scale (for depression) (K-CES-D) and Connor-Davidson Resilience Scale-Korea (CD-RI-K) (for self-resilience) were used.

#### Korean center for epidemiologic studies depression scale (K-CES-D)

To measure depression, which was expected to be associated with PTSD symptoms, the adapted Korean version of the Center for Epidemiologic Studies Depression Scale (CES-D) was used. It consisted of 20 questions based on symptoms of depression that were experienced during the week; the answers ranged on a scale from 0 to 3 where 0, 1, 2, and 3 denoted very rarely, rarely, sometimes, and most of the time, respectively. Questions 4, 8, 12, and 16, where positive meanings were contained, were reversely calculated. Total score below 16, 16–20, 21–24, and over 25 indicated normal, mild, severe, and very severe, respectively. The reliability and validity of this questionnaire are well established [12].

#### CD-RI-K

To measure self-resilience, a Korean-version of the self-resilience scale (CD-RISC-K), which is an adapted version of CD-RISC [13] developed by Block and Klomen, was used. The scale included 25 questions in five areas regarding toughness, durability, optimism, support, and spirituality as subareas with a scale of 0 to 4, where 0, 1, 2, 3, and 4 represented absolutely not, not, usually, yes, and absolutely yes, respectively. The range of scores was 0 to 100. A higher score indicated higher resilience. In this study, the total scores were analyzed as continuous variables. In addition, based on the average score for self-resilience (64.5) of 552 firefighters in Korea in a previous study [14], two groups were formed for analysis: low resilience and high resilience groups. Several studies have established this questionnaire as a reliable and valid instrument for measuring symptoms of depression in the Korean population [15].

### Critical incident exposure

Through a self-survey, we investigated the extent of exposure to traumatic events such as a disaster, an accident, physical, or sexual abuse (“experienced,” “witnessed,” “learned of,” “not sure,” and “doesn't apply”) while working. Those who experienced or witnessed a traumatic event while working were included in the final analysis.

### Current PTSD symptoms

PTSD symptoms were measured using the Impact of Event Scale-Revised-Korean (IES-R-K).

#### IES-R-K

The IES-R-K is a 22-item self-reporting method that assessed subjective distress caused by traumatic events. Items corresponded directly to 14 of the 17 Diagnostic and Statistical Manual of Mental Disorders, 4th Edition (DSM-IV) symptoms of PTSD. Respondents were asked to identify a specific stressful life event, and then, indicate how much they were distressed or bothered during the past 7 days by each “difficulty” listed. Items were rated on a 5-point scale ranging from 0 (“not at all”) to 4 (“extremely”). The IES-R yields a total score (ranging from 0 to 88), where IES-R score ≤24 = normal, 25–39 = mild/moderate symptoms of PTSD, 40–59 = severe symptoms of PTSD, and ≥60 = very severe symptoms of PTSD. In this study, anyone with 25 points or more based on IES-R-K score was defined as having PTSD symptoms.

### Statistical analysis

To analyze the results, the chi-squared test, two-sample *t*-test, and logistic regression were used. We conducted a descriptive analysis on the general and occupational characteristics of the subjects surveyed. The crude odds ratios (OR) between the expected protective factors, that is, self-resilience and occurrence of PTSD symptoms and 95 % confidence intervals (95 % CI) were calculated using a simple logistic regression analysis. To identify the factors that might influence PTSD symptoms, factors were corrected for and were applied as independent variables, and PTSD symptoms were applied as the dependent variable in the logistic regression analysis. Model 1 was based on crude OR; model 2 included the socio-demographic characteristics as the independent variables and model 3 included the socio-demographic characteristics and occupational factors as the independent variables. Model 4 included the socio-demographic characteristics, occupational factors, and depression as the independent variables. OR and 95 % CI were calculated. All data were analyzed using SPSS version 22.0.

## Results

### General characteristics of the study subjects

The age of study subjects ranged from 49 to 60 years (M = 54.40, SD = 3.27). Regarding the level of education completed, those with college-level education or above constituted the largest proportion (50.0 %, 56), followed by those with high-school diploma (39.3 %, 44), and middle school or below (10.7 %, 12). Regarding the participants’ smoking status, 45.5 % (51) were non-smokers, 42.9 % (48) were current smokers, and 11.6 % (13) were ex-smokers. Among those surveyed, 53.6 % (60) drank ≥ two alcoholic drinks a week, while 25.0 % (28) drank ≤ one drink a week. Non-drinkers represented 21.4 % (24) of the total subjects. Regarding occupational characteristics, 58 % (65) were city patrollers, and 41.9 % (47) were rural patrollers. Further, 23.2 % (26) had worked for more than 10 and less than 20 years; however, 35.7 % (40) had worked for more than 20 years. Based on the reference values of occupational stress for men (Short Form) [11], the whole group was categorized by quartile to classify their job stress; it was observed that 33.9 % (38 persons) belonged to Q2, and 27.7 % (31) belonged to Q3. More than 55.4 % (62) of the respondents had depression (above mild). In this study, 33.9 % (38) had low self-resilience below 64.5 points. Moreover, 18 out of 112 showed PTSD symptoms.

### Association between general characteristics and PTSD symptoms

On analysis of the usual, occupational, and psychological characteristics according to the prevalence of PTSD symptoms, we expected that police officers working in urban areas might have higher prevalence rate of PTSD symptoms, owing to a higher exposure intensity and frequency to criminal cases; however, there was no significance. We observed that higher job stress (*p* = 0.019) and depression (*p* = 0.007) and lower self-resilience (*p* < 0.001) led to higher prevalence rate of PTSD symptoms (Table 1).

**Table 1:** Association between general characteristics and PTSD symptoms (*n* = 112)

General characteristics	PTSD symptoms				*p*-value*
	Yes (*n* = 18)		No (*n* = 94)		
	*n*	%	*n*	%	
Age (years)^a^	54.44 ± 3.27		54.06 ± 3.28		0.65
Level of education completed					
Middle school or below	2	11.11	10	10.64	0.87
High school	8	44.44	36	38.30	
College or above	8	44.44	48	51.06	
Marital status					
Married	16	88.89	85	90.43	0.56
Divorced or widowed	2	11.11	9	9.57	
Smoking					
Non-smoker	8	44.44	43	45.74	0.76
Ex-smoker	3	16.67	10	10.64	
Current smoker	7	38.89	41	43.62	
Alcohol drinking frequency (drinks)					
None	4	22.22	20	21.28	0.94
≤ 1 per week	5	27.78	23	24.47	
≥ 2 per week	9	50.00	51	54.25	
Service area (for patrol)					
Rural	6	33.33	41	43.62	0.29
Urban	12	66.67	53	56.38	
Duration of patrol service (years)					
10<	8	44.44	38	40.43	0.95
10–19	4	22.22	22	23.40	
≥ 20	6	33.33	34	36.17	
Job stress^a^	57.25 ± 12.06		45.57 ± 10.91		0.02
Depression					
Normal	2	11.11	48	51.06	0.01
Mild	6	33.33	26	27.66	
Severe	6	33.33	12	12.77	
Very severe	4	22.22	8	8.51	
Self-resilience^b^					
High (CD-RI-K score ≥64.5)	6	33.33	68	72.34	0.00
Low (CD-RI-K score <64.5)	12	66.67	26	27.66	
Self resilience^a^·^c^	51.93 ± 8.21		69.58 ± 8.67		0.00
(Total CD-RI-K score)					

Based on a chi-squared test*

^a^and two-sample *t*-test

^b^Categorical data 1: Data comparing two groups (64.5 was a standard value)

^c^Continuous data 2: Continuous comparison data based on the total score of K-CDRI; higher score means higher resilience

### Odds ratios of PTSD symptoms associated with self-resilience

Self-resilience was observed to have a significant association with PTSD symptoms. We set 64.5 as a standard value and analyzed self-resilience by classifying the subjects into low self-resilience and high self-resilience groups. For the low self-reliance group, crude OR and 95 % CI of PTSD symptoms were 4.23 and 1.79–15.39, respectively. The OR and 95 % CI of models 2, 3, and 4 after correcting for the general, occupational, and psychological characteristics were 4.80 and 1.88–17.96, 4.98 and 1.73–21.40, and 3.51 and 1.06–19.23, respectively. Based on this, we could ascertain that the low self-resilience group had higher prevalence of PTSD symptoms. When the total score of self-reliance was analyzed by using continuous data, it was observed that higher self-reliance was negatively related to the occurrence of PTSD symptoms. Model 1 (OR, 0.90; 95 % CI, 0.83–0.94), model 2 (OR, 0.87; 95 % CI, 0.83–0.93), model 3 (OR, 0.77; 95 % CI, 0.65–0.91), and model 4 (OR, 0.74; 95 % CI, 0.58–0.93) are represented in (Table 2). Using continuous data of total resilience score also showed that higher self-resiliency might lead to lower prevalence of PTSD symptoms even after adjusting for the co-variables.

**Table 2:** Odds ratios of PTSD symptoms associated with self-resilience

	Model 1	Model 2	Model 3	Model 4
	OR 95 % CI	OR 95 % CI	OR 95 % CI	OR 95 % CI
PTSD symptoms				
Reference group	1.00	1.00	1.00	1.00
Low self-resilience^a^	4.23 1.79–15.39	4.80 1.88–17.96	4.98 1.73–21.40	3.51 1.06–19.23
(CD-RI-K score <64.50)				
Total CD-RI-K score^b^	0.90 0.83–0.94	0.87 0.83–0.93	0.77 0.65–0.91	0.74 0.58–0.93

Model 1: Odds ratio by independent univariate logistic regression analysis

Model 2: Multivariate logistic regression analysis, model 1 + age, level of education completed (middle school or below, high school, or college or above), marital status (married, divorced, or widowed), smoking (non-smoker, ex-smoker, or current smoker), and drinking frequency (none, ≤1 per week, ≥2 per week)

Model 3: Multivariate logistic regression analysis, model 2 + service area for patrol (rural or urban), duration of patrol service (<10 years, 10–19 years, or ≥20 years), and job stress

Model 4: Multivariate logistic regression analysis, model 3 + depression (normal, mild, severe, or very severe)

*Abbreviations*: *OR* odds ratio, *CI* confidence interval

^a^Categorical data 1: Data comparing two groups (64.5 was a standard value)

^b^Continuous data 2: Continuous comparison of data based on the total scores of K-CDRI; a higher score means higher resilience

## Discussion

Although exposure to trauma is common, PTSD symptoms and other mental disorders after trauma are relatively rare. In order to allow early intervention, the core methodological objective of research on traumatic stress is to determine factors that might have a role in psychopathological symptoms leading to PTSD symptoms. These efforts are particularly essential for professional populations at high risk for trauma-related disorders (for example, police officers, firefighters, and military personnel who are regularly engaged in traumatic events). The present study aimed to identify the mechanisms underlying the interactions among risk and protective factors (such as self-resilience) related to PTSD symptoms. This study suggested that the association between traumatic exposure and PTSD symptoms might be moderated by an individual’s resilience. The results of the multivariate logistic regression analysis show that a low level of self-resilience is a strong predictor of development of PTSD symptoms in police officers who are exposed to traumatic events.

The present data support and extend the clinical evidence regarding the role of personality traits in PTSD symptoms. In the study, self-reliance scores were processed continuously, and it was observed that self-reliance was negatively correlated with the occurrence of PTSD symptoms. We also analyzed CD-RI-K score categorized into four and five groups in each and found no significance. Based on the average score of firefighters from an existing study (CD-RI-K score = 64.5), self-reliance was categorized into two groups. The low self-resilience group whose CD-RI-K score was below 64.5 had a high occurrence rate of PTSD symptoms. In the present study, participants with PTSD symptoms showed low self-reliance scores similar to a previous study based on firefighters. Many studies have examined the protective or predictable factors for PTSD symptoms [16–18]. Several studies have shown that not all individuals exposed to a trauma develop PTSD symptoms and that some are able to manage and cope with adversity; such findings indicate the importance of resilience as a protective factor [19, 20]. Resilience can be defined as the ability to adapt and successfully cope with acute or chronic adversity. Resilience reduces susceptibility to depression and suicide in individuals with childhood trauma [21, 22] and in veterans [23]. Furthermore, lower levels of self-efficacy have previously been related to PTSD symptoms [24–26]. Lower self-efficacy levels were observed in individuals with PTSD symptoms compared to healthy subjects; however, traumatized individuals without PTSD symptoms did not differ in self-efficacy levels compared to healthy subjects [26]. These findings illustrate the importance of individual’s resilience as a protective factor against PTSD symptoms in high-risk groups; however, recent models of resilience include risk as well as protective factors that may interact to reduce negative consequences and facilitate positive ones [27]. In this context, a better understanding of pre-traumatic risk factors would certainly have important clinical implications regarding the development of trauma-related disorders. Self-resilience is known to be one of the buffering factors for personal stressors, but might also play a leading role in preventing PTSD symptoms, if under organized control. Although not analyzed in this study, self-resilience can be developed by interventions in the workplace through communication, mutual respect, and dissolution of interpersonal conflict. Further prospective, longitudinal design studies examining resilience/intervention in the workplace are needed.

This study has several limitations that should be noted. First, the cross-sectional design of this study limits its ability to confirm a causal relationship among the variables that were investigated. Thus, the results of the present study should be interpreted cautiously, and future research using longitudinal and experimental designs is needed to clarify the direction of any causal relationships among the observed variables. Second, the present research relied solely on self-reported questionnaires. Although we have conducted the study on those who have not experienced traumatic events for the past 6 months and without any underlying mental disorders, we could not exclude subjects who might have had mild symptoms of PTSD already or short duration (less than 1 month) of PTSD symptoms. Moreover, we have not assessed the characteristics of their exposure: intensity, interval, and frequency of critical incidents. Future studies utilizing standardized interviews or observational methods would provide a more accurate and detailed information regarding the complex mechanisms underlying the relationship between traumatic stress and development of PTSD symptoms. Third, we could not consider multiple mechanisms involving protective psychological factors (for example, social support and self-efficacy), other personality factors, and possible underlying neurobiological mechanisms (for example, neuropeptide oxytocin), which could influence PTSD symptoms. There were also some elements missing from the analysis for the occupational factors, our survey did not include the exact type of job title, department, the frequency of emergent dispatch, case experience, and case types, and thus, this information was not reflected in the study. There were limitations associated with not taking into account the aforementioned factors, which can have a major impact on onset of PTSD symptoms. Finally, the present study included only a few participants. For this reason, future studies with a large number of participants are needed to achieve statistical significance for generalization of the current results. However, 2015 was the first year of our study based on police officers; they will be followed up for annual medical screening and examination, and we plan to conduct a cohort study on the health of police officers in Gangwon. Further, we will investigate various factors, which could influence PTSD symptoms. Future longitudinal studies will be conducted that include a large number of study subjects.

## Conclusions

Despite the limitations, to our best knowledge, the current study is the first to identify the protective effects of self-resilience against the development of PTSD symptoms in police officers after adjusting for occupational characteristics. Although the reported results need to be replicated to draw concrete conclusions, self-resilience as a protective factor might eventually help clinicians and specific organizations to target individuals who are at a high risk of developing trauma-related disorders (for example, police officers, firefighters, and military personnel). In particularly, additional studies on police officers would be actively pursued based on the fact that for general occupational groups those have low exposure intensity, data from studies on police officers may actually be useful. Identification of protective or risk factors might provide clues regarding the underlying mechanisms and could help in building new strategies to prevent the development of a disorder [28]. Finally, the results might indicate that a coping skill training (for example, self-resilience training) could be helpful for primary and secondary prevention in populations at high risks.

## Abbreviations

CD-RI-K: Connor-Davidson Resilience Scale-Korean
CES-D: Center for Epidemiologic Studies-Depression Scale
DSM-IV: Diagnostic and Statistical Manual of Mental Disorders, 4th Edition
IES-R-K: Impact of Event Scale-Revised-Korean version
KOSS-SF: Korean Occupational Stress Scale-Short Form
OR: Odds ratio
PTSD: Post-traumatic stress disorder

## References

[1] Carlier IV, Lamberts RD, Gersons BP (1997). Risk factors for posttraumatic stress symptomatology in police officers: A prospective analysis. J Nerv Ment Dis.

[2] Foa EB, Hearst-Ikeda D, Perry KJ (1995). Evaluation of a brief cognitive-behavioral program for the prevention of chronic PTSD in recent assault victims. J Consult Clin Psychol.

[3] Robinson HM, Sigman MR, Wilson JP (1997). Duty-related stressors and PTSD symptoms in suburban police officers. Psychol Rep.

[4] Marmar CR, McCaslin SE, Metzler TJ, Best S, Weiss DS, Fagan J, et al. Predictors of posttraumatic stress in police and other first responders. Ann N Y Acad Sci. 2006;1071:1–18. 10.1196/annals.1364.001 16891557

[5] Rutter M (1985). Resilience in the face of adversity: Protective factors and resistance to psychiatry disorder. Br J Psychiatry.

[6] Han YM. Relationships between self resilience and psychological health in college students. Seoul, Korea: Doctoral thesis of Hongik University Graduate School; 2009. vol. 30, p. 97–110.

[7] Caver CS, Scheier MF (1991). Unresolved issues regarding the meaning and measurement of explanatory style. Psychol Inq.

[8] Brewin CR, Andrews B, Valentine JD (2000). Meta-analysis of risk factors for posttraumatic stress disorder in trauma-exposed adults. J Consult Clin Psychol.

[9] Mcnally RJ (2003). Psychological mechanisms in acute response to trauma. Biol Psychiatry.

[10] Yuan C (2011). Protective factors for posttraumatic stress disorder symptoms in a prospective study of police officers. Psychiatry Res.

[11] Chang SJ. Standardization of job stress measurement scale for Korean employee. Incheon: Korea Occupational Safety and Health Agency. 2004;17–41. Korean.

[12] Cho MJ, Kim KH (1998). Use of Center for Epidemiologic Studies Depression (CES-D) scale in Korea. J Nerv Ment Dis.

[13] Connor KM, Davidson JRT (2003). Development of a new resilience scale: The Connor-Davidson Resilience scale (CD-RISC), depression, and anxiety. Depress Anxiety.

[14] Lee JS (2014). Resilience buffers the impact of traumatic events on the development of PTSD symptoms in firefighters. J Affect Disord.

[15] Jung YE (2012). The Korean version of the Connor-Davidson Resilience Scale: An extended validation. Stress Health.

[16] Heinrichs M (2005). Predicting posttraumatic stress symptoms from pre-traumatic risk factors: a 2 year prospective follow-up study in firefighters. Am J Psychiatry.

[17] David S (2014). Trauma exposed firefighters; Relationships among posttraumatic growth, posttraumatic stress, resource availability, coping and critical incident stress debriefing experience. Stress Health.

[18] Bryant RA, Guthrie RM (2007). Maladaptive self appraisals before trauma exposure predict posttraumatic stress disorder. J Consult Clin Psychol.

[19] Agaibi CE, Wilson JP (2005). Trauma, PTSD, and resilience: A review of the literature. Trauma Violence Abuse.

[20] Hoge EA, Austin ED, Pollack MH (2007). Resilience: Research evidence and conceptual considerations for posttraumatic stress disorder. Depress Anxiety.

[21] Roy A, Carli V, Sarchiapone M (2011). Resilience mitigates the suicide risk associated with childhood trauma. J Affect Disord.

[22] Wingo AP, Wrenn G, Pelletier T, Gutman AR, Bradley B, Ressler KJ (2010). Moderating effects of resilience on depression in individuals with a history of childhood abuse or trauma exposure. J Affect Disord.

[23] Pietrzak RH, Johnson DC, Goldstein MB, Malley JC, Rivers AJ, Morgan CA (2010). Psychosocial buffers of traumatic stress, depressive symptoms, and psychosocial difficulties in veterans of Operations Enduring Freedom and Iraqi Freedom: The role of resilience, unit support, and postdeployment social support. J Affect Disord.

[24] Benight CC, Harper ML (2002). Coping self-efficacy perceptions as a mediator between acute stress response and long-term distress following natural disasters. J Trauma Stress.

[25] Benight CC, Antoni MH, Kilbourn K, Ironson G, Kumar MA, Fletcher MA (1997). Coping self-efficacy buffers psychological and physiological disturbances in HIV-infected men following a natural disaster. Health Psychol.

[26] Saigh PA, Mroueh M, Zimmerman BJ, Fairbank JA (1995). Self-efficacy expectations among traumatized adolescents. Behav Res Ther.

[27] Fergus S, Zimmerman MA (2005). Adolescent resilience: a framework for understanding healthy development in the face of risk. Annu Rev Public Health.

[28] Kraemer HC, Stice E, Kazdin A, Offord D, Kupfer D (2001). How do risk factors work together? mediators, moderators, and independent, overlapping, and proxy risk factors. Am J Psychiatry.

